# Clinician and Patient Perspectives on the Exchange of Sensitive Social Determinants of Health Information

**DOI:** 10.1001/jamanetworkopen.2024.44376

**Published:** 2024-10-31

**Authors:** Catherine M. DesRoches, Deborah Wachenheim, Annalays Garcia, Kendall Harcourt, JaWanna Henry, Ria Shah, Vaishali Patel

**Affiliations:** 1 Department of Medicine, Harvard Medical School, Boston, Massachusetts; 2 OpenNotes, Division of General Medicine, Beth Israel Deaconess Medical Center, Boston, Massachusetts; 3 Department of Health and Human Services, Assistant Secretary for Technology Policy and Office of the National Coordinator for Health Information Technology, Washington, DC

## Abstract

**Importance:**

Social determinant of health (SDOH) data are essential to individualized care and reducing health disparities. However, there is little standardization in the way that SDOH data are collected, and barriers to increasing the collection of such data exist at both the patient and clinician levels.

**Objective:**

To evaluate clinician, patient, and care partner perspectives on the barriers to and facilitators of patients sharing SDOH information with their clinicians.

**Design, Setting, and Participants:**

This qualitative study included clinicians, patients, and care partners across the United States. Focus groups were conducted between September 2022 and February 2023 to understand the experience of collecting, documenting, and exchanging SDOH data.

**Main Outcomes and Measures:**

Rapid assessment procedures were used to analyze focus group transcripts, creating summaries, codes, and themes mapped directly to the project research questions.

**Results:**

A total of 235 individuals participated, including 109 (46.4%) clinicians (60 [55.0%] male; 25 [22.9%] Asian, 2 [1.8%] Black, and 74 [67.9%] White) and 126 (53.6%) patients and care partners (45 [35.7%] male; 1 [0.8%] Asian, 48 [38.1%] Black, and 64 [50.8%] White). Clinicians and patients agreed that SDOH data are important for clinicians to know. Both clinicians and patients wanted a structured, standardized way to collect SDOH data in the future, accompanied by time for more in-depth discussion during the visit. However, they highlighted numerous issues that impact collecting these data, including beliefs about how the information will be used, the clinician-patient relationship, having enough of the right staff, time needed to collect SDOH information, and technology used to collect the data (eg, usability, standardization).

**Conclusions and Relevance:**

This qualitative study of the experience of collecting, documenting, and exchanging SDOH data underscores the ongoing barriers to widespread adoption of uniform approaches to SDOH data documentation as well as factors that may help lower those barriers, such as trusting patient-clinician relationships, greater transparency in how the data will be used, and targeted resources. A multifaceted approach to addressing the concerns raised by clinicians, patients, and care partners is required to ensure that such data can be captured in a way that improves care and allows for progress toward an equitable health care system.

## Key Points

**Question:**

What are the barriers to and facilitators of the collection, documentation, and exchange of social determinants of health (SDOH) data among clinicians, patients, and care partners?

**Findings:**

This qualitative study of 126 patients and care partners and 109 clinicians found that while there was widespread agreement about the importance of collecting SDOH data, there were multiple barriers to its effective collection, documentation, and exchange. Patients and care partners expressed concerns about how the information is utilized, although a trusting patient-clinician relationship ameliorated that concern, and clinicians reported that they often feel they have limited time and resources to put toward best assisting patients.

**Meaning:**

The findings of this study suggest that a multifaceted approach, taking both patients’ and clinicians’ concerns and preferences into account, is needed to improve the collection, documentation, and exchange of SDOH data to benefit both direct patient care and broader efforts at improving public health.

## Teaser

This qualitative study engages with clinicians, patients, and care partners to better understand the experience of collecting, documenting, and sharing social determinant of health data.

## Introduction

National efforts are underway to increase and standardize the documentation of social determinants of health (SDOH)—nonmedical factors that influence health outcomes, including conditions in which people are born, grow, work, live, and age—which may account for 50% to 80% of health outcomes.^1,2,3^ SDOH data are part of the US Core Data for Interoperability (USDCI), a standardized set of data classes and elements for nationwide, interoperable health information exchange.^4,5,6^ The Centers for Medicare & Medicaid Services (CMS) have supported care delivery models, including the Accountable Health Communities (AHC) model, requiring the collection of standardized SDOH data.^7^ Medicaid programs are increasingly requiring SDOH documentation as a quality metric for Medicaid accountable care organizations.^8^ Private insurers are calling for collection of data necessary to identify and ameliorate health disparities.^9,10^

Despite growing recognition of SDOH’s effect, its routine and structured documentation using uniform approaches is not widespread.^11,12,13,14,15,16^ The lack of standardization has led to a multitude of screening tools, standardized terminologies, and differences in how and what is documented, despite agreement among clinicians of the data’s importance.^11,12,17,18,19,20,21,22^

Patients’ experiences and perspectives on collecting and sharing SDOH data are not well understood. The limited literature suggests that while patients agree information about SDOH is important for clinicians to know, they have concerns about it being documented in the medical record.^23,24,25,26^ Studies on the effect of sharing clinical notes with patients suggest there are benefits; however, approximately 1 in 10 patients feel offended by something they read in a note, often a personal descriptor or sensitive information.^27^ Given the sensitive nature of many SDOH elements, it is important to understand how patients view the need and acceptability of having it in the EHR, where it can be accessed by multiple clinicians.

We conducted focus groups to understand how patients and clinicians are experiencing the increasing emphasis on collecting and documenting SDOH. We sought to address 3 questions. First, what barriers to widespread and standardized documentation of SDOH data do clinicians and patients experience? Second, what do they think could facilitate data collection? And, finally, what are their preferences for future data collection?

## Methods

### Focus Group Guides

We completed an environmental scan of the literature on SDOH documentation practices in the United States and on patient and clinician perspectives (eAppendix 1 in Supplement 1). We next created focus group guides that underwent multiple rounds of revision with our expert advisory panel (EAP) comprising clinicians, patients, and subject matter experts (eAppendix 2 in Supplement 1). The guides are available from the authors on request.

The project was approved by the Institutional Review Board for Human Subject Research at Beth Israel Deaconess Medical Center. The study was declared exempt from the requirement for informed consent by the institutional review board. Participants were informed through a prospective agreement about their rights as research participants and told that data, including quotations, would be used for research. We adhered to the Standards for Reporting Qualitative Research (SRQR) reporting guideline to ensure transparency, rigor, and comprehensiveness. These guidelines informed our study design, data analysis, and reporting.^28^

### Patient and Clinician Groups

We developed the list of patient and clinician groups to be as representative as possible (Table 1). We contracted with a market research firm to (1) recruit participants from their national database, (2) moderate the focus groups using synchronous and asynchronous online platforms, and (3) provide transcripts for all groups.

**Table 1.** Number of Focus Groups

Groups	Groups, No. (%)^a^	Participants, No. (%)^a^
Health care clinician groups		
Total, No.	10	109
Primary care	2 (20.0)	22 (20.2)
Pediatrics	2 (20.0)	22 (20.2)
Mental health	1 (10.0)	11 (10.1)
Specialty care	1 (10.0)	11 (10.1)
Emergency medicine	1 (10.0)	11 (10.1)
Case management and social work	1 (10.0)	11 (10.1)
Practitioners serving rural or underserved areas or safety-net practitioners	2 (20.0)	21 (19.3)
Patient groups		
Total, No.	10	126
Black or African American patients	2 (20.0)	27 (21)
Spanish-speaking patients	1 (10.0)	14 (11.1)
Parents or guardians of children <18 y	1 (10.0)	12 (9.5)
Individuals with disabilities or multiple chronic health conditions	1 (10.0)	11 (8.7)
Patients ≥65 y	1 (10.0)	14 (11.1)
Individuals in underserved or low-income areas	1 (10.0)	12 (9.5)
Individuals living in rural areas	1 (10.0)	13 (10.3)
LGBTQ+	1 (10.0)	13 (10.3)
Patient advocates	1 (10.0)	10 (7.9)
Total, No.	20	235

Abbreviation: LGBTQ+, lesbian, gay, bisexual, transgender, queer, and other minoritized sexual or gender identities.

^a^ Percentages are calculated by group (ie, clinicians or patients and care partners).

#### Clinicians

The firm recruited participants for 10 clinician groups from their database of individuals willing to be contacted for research. The groups encompassed: primary care (physicians [MDs], advanced nurse practitioners [ANPs], and physician assistants [PAs]); clinicians working in pediatrics (MDs, ANPs, and PAs); mental health clinicians (including social workers and psychologists); specialists (including MDs and occupational and physical therapists); emergency medicine (including emergency medical technicians); primary care practitioners (PCPs) practicing in rural communities; PCPs working in underserved urban areas; and case managers (including licensed social workers and registered nurses) (Table 1). Practicing in one of these specialties was the primary inclusion criteria. Clinicians were paid $200 for their participation. eAppendix 5 in Supplement 1 presents participant characteristics.

These groups were conducted using an asynchronous virtual discussion board platform over a period of 3 days per group.^29^ Research presents a mixed picture of the effectiveness of asynchronous groups. Some findings suggest that the method provides more in-depth information and allows participants to be thoughtful and comprehensive, while others note the lack of conversational nuance.^30,31^ We chose this method to accommodate the schedules of busy clinicians. Invited participants logged on to the platform to answer questions at their own pace. Participants could respond to each other’s answers, and the firm’s professional moderator was able to ask follow-up questions.

#### Patients

The firm recruited patients from the following groups: persons self-identifying as Black or African American; persons with Spanish as their preferred language; parents or guardians of children younger than 18 years; persons with disabilities or multiple chronic conditions; persons aged 65 years or older; persons living in underserved urban areas; persons living in rural areas; persons identifying as lesbian, gay, bisexual, transgender, queer, and other sexual and gender minority groups (LGBTQ+); and patient advocates (Table 1). Patients and care partners self-identified as meeting the eligibility criteria and were paid $100 for their participation.

Nine patient groups were conducted synchronously using an online video conferencing platform. One patient group (patient advocates) was conducted using the asynchronous method. Because patients and care partners may have limited knowledge of SDOH, the moderator introduced the topic first by giving the US Centers for Disease Control and Prevention definition of SDOH^32^ and then asking the participants for examples of what could be included in that definition. After a brief discussion the moderator then showed a set of slides to give more information about SDOH. This background informed the rest of the discussion (eAppendix 3 in Supplement 1).

All groups were conducted between September 2022 and February 2023. All synchronous groups were professionally moderated, recorded, translated (Spanish language groups), and transcribed. Transcripts from the asynchronous groups were provided in spreadsheet form.

### Data Analysis

Rapid assessment procedures (RAPs) were used to analyze the focus group transcripts. This flexible but rigorous approach to qualitative data analysis is appropriate for studies conducted over a short time frame with a small number of research questions.^33,34,35^ Following the principles of RAP, we created an a priori set of codes mapped directly to our research questions to identify barriers, facilitators, and preferences for future data collection. Two members of the research team (K.H. and A.G.) independently summarized the first clinician and patient transcripts to align the summarization process, resolving discrepancies through discussion. Remaining group transcripts were summarized by one team member. Four members of the project team (K.H., A.G., D.W., and C.M.D.) served as transcript coders. Two coders (K.H. and A.G.) reviewed the first transcripts for the patient and clinician groups and independently verified that our initial codes were appropriate, adding new codes as necessary. These 2 coders met to discuss discrepancies and created a harmonized code list. Each transcript was then coded by 2 coders. The list of codes appears in eAppendix 4 in Supplement 1. Discrepancies between the 2 coders that could not be resolved through discussion were adjudicated by a senior member of the research team (C.M.D.). The coded transcripts were compared with the initial summaries as an additional level of review for consistency.

The 31 codes were next grouped into 6 themes relating to factors affecting data collection: (1) beliefs about how the information will be used, (2) patient-clinician relationship, (3) structural issues, (4) workflow-related issues, (5) technical issues, and (6) policy options. Two additional themes related to preferences for future data collection: (1) collect/provide information through conversation and (2) collect/provide data via structured questionnaires. Quotes that are illustrative of each theme were extracted from the transcripts and reviewed by the EAP.

## Results

A total of 217 clinicians and 133 patients completed the prescreening survey to determine eligibility, and 109 clinicians (60 [55.0%] male; 25 [22.9%] Asian, 2 [1.8%] Black, and 74 [67.9%] White) and 126 patients and care partners (45 [35.7%] male; 1 [0.8%] Asian, 48 [38.1%] Black, and 64 [50.8%] White) were eligible and participated in the groups. eAppendix 5 in Supplement 1 presents participant characteristics.

### How SDOH Data Are Used

Clinicians and patients agreed that SDOH data are important for clinicians to know. However, they highlighted numerous factors that affect the standardized collection of this data (Table 2).

**Table 2.** Clinician- and Patient-Identified Issues Affecting SDOH Data Collection and Documentation

Themes	Examples (group)
How the information will be used	“I do sometimes wonder, they’re asking these questions but also do they have the, hopefully they also have the training to handle not, that not biasing their opinions about the patient because that is, that information can change sometimes how you think about people. So it makes you just kind of—like I’m OK with sharing it but also it makes me wonder like how will they receive it?” (Parents or guardians of children <18 y) “Providers unwilling to utilize the information… unable to see the value of the information. This is concerning as either of these behaviors could render a treatment ineffective.” (Specialty care) “I think we probably just need to change the culture around how I feel like it’s being perceived by the doctor. If I felt like I had a doctor who would read that and never not listen to me because of it or something like that, then I’m down for him to know… but I think it’s when I feel like it’s being used almost against me or to not give me the help that I need, that’s when it’s a problem.” (Black or African American patients)
Clinician-patient relationship	“I went through a rough spot, like what I’m going through right now, I don’t want that to impact anybody I might see, in the future… I don’t want them looking at me sideways because I had a difficult patch.” (Patients with disability) “Bringing up some of these issues and how it affects a patient’s care can be awkward sometimes if it is in regards to race or sexual orientation, because I don’t want to offend anyone by talking about those things.” (Rural primary care) “And I think it’s because of the doctor that I do have now that I am more open because we built that relationship… But it’s like where I am now, I trust my doctor, so I do open up and share.” (Black or African American patients)
Workflow	“The main issue is that if we collect the info, we wouldn’t have enough time built into the day to act on the information gathered or provide meaningful help to the patient.” (Pediatrics) “I just feel like I brought this up earlier like I’m just another number they try to get you out so fast so they can go to the next appointment or the next client.” (Black or African American patients) “More time for providers to do their work! SDOH, while important, suffers from the add-on phenomenon... From suicide risk screening to HIV risk screening, the amount of questions required/mandated is exhaustive and incredibly time consuming.” (Emergency medicine)
Structural	“We can collect the information, but then we do not have the next step in place to help the patients. Giving them a list of resources doesn’t work, since then they need to take the step of calling or contacting an agency or group.” (Pediatrics) “[Participant would need] embedded behavioral health professionals in primary care settings WITH adequate time to be a part of the team.” (Rural primary care) “I think it would be helpful to have more access to resources for other agencies who do specialize in the areas of SDOH that are out of my scope. I feel I address the needs of food insecurity pretty well, but I don’t know where or how to connect a patient with resources for domestic violence or gender identity questions.” (Specialty care)
Technology	“Information overload! Reviewing old medical records should be helpful, but most EHR systems collect so much useless jargon and documentation that the EHR becomes diluted and less valuable as a tool.” (Emergency medicine) “I have a kind of fear of computers and technology because people hack everything and you’d never know who’s trying to find out what’s going on about you.” (Patients ≥65 y)
Policy	“I think most important would be funding to support staff to systematically collect and address these issues. Doctors and some other providers are usually good at addressing these issues when they have adequate time, but that is not the norm, to have adequate time.” (Rural primary care) “If the government mandated a certain way of doing things and gave a monetary reward for doing it, that would promote its use. I am currently not aware of any policies.” (Urban primary care) “I think if medical teams were educated on the importance of this information - it would impact more documentation. It is part of a Social Workers practice to document SDOH, if there is a way to ensure this is happening more consistently through an assessment, it would be lead to more documentation.” (Mental health)

Abbreviations: EHR, electronic health record; SDOH, social determinants of health.

Patients and clinicians noted concerns about how SDOH information could be used as a barrier to data collection. These included differences in knowledge of data collection and use between patients and clinicians, creating misunderstanding or communication issues and concerns about data privacy. Both expressed uncertainty about the value of the information for purposes beyond informing clinical care. While clinicians reported using the data for individual treatment and referral decisions, most did not know whether their health care organization was using it for population health management.

Patients expressed an additional barrier—the fear that sharing SDOH data could result in discrimination or bias. They expressed reluctance to answer SDOH questions and suggested that it could be used to negatively affect or limit their care. Patients expressed particular concern about sharing financial information, where they live, and their level of education. This concern was prominent in the groups of Black patients, patients with disabilities, the LGBTQ+ group, and among Spanish speakers.

Many clinicians also expressed the concern that patients would worry about sensitive information being used against them. In addition, clinicians reported worries about getting SDOH data from other sources, such as clinicians from other organizations, social service agencies, and schools. They noted that it is filtered through the perceptions of the documenting person and may reflect assumptions and unspoken biases.

### Clinician-Patient Relationship

Clinicians and patients agreed that a poor patient-clinician relationship could hinder the disclosure of SDOH data (Table 2). Clinicians reported discomfort with asking patients these sensitive questions, either because they were uncomfortable or thought that patients would be offended. Patients also noted that clinicians might be uncomfortable with these conversations.

Alternatively, patients identified a trusting relationship with a clinician as positively affecting their willingness to have SDOH data documented. Patients generally felt comfortable sharing SDOH data with, and having their information recorded by, their PCP. They felt less comfortable discussing or having it shared with other clinicians. Patients suggested the following changes would increase their willingness to share information: patient-granted permission for sharing specific information with other clinicians; documenting and sharing only information the patient believed was relevant; and the ability for patients to electronically share information directly with clinicians of their choice.

### Structural and Workflow Issues

Numerous structural and workflow barriers were raised, particularly by clinicians. Both clinicians and patients were reluctant to discuss SDOH-related factors if the practice did not have resources to address the identified needs. Clinicians consistently noted lack of time as a significant barrier for gathering and discussing SDOH data with patients and assisting with resources and referrals. Clinicians described patients with other pressing clinical needs and understaffing as contributors to the lack of time available to focus on SDOH. Patients also identified time as a barrier, noting that clinicians are often rushed during visits.

Clinicians discussed the importance of having trained staff who can focus on SDOH and assist patients. Without this availability, those involved in the data collection and documentation may not be aware of the importance of SDOH or may lack the skills to discuss these topics. They noted that trained staff also would be more likely to know about resources and referrals that could assist the patients and would know how to build rapport and cultivate trust.

### Technology-Related Issues

Clinicians and patients identified aspects of technology that impacted data collection. Some clinicians reported that it may not be clear where to document SDOH data in the EHR and how to easily retrieve it. Clinicians also commented on the lack of a standard method to collect data in the EHR. Patients noted lack of access to or comfort with technology as an additional barrier. Finally, patients expressed a general concern about the digital safety of their electronic data.

### Policy and Organizational Issues

Clinicians suggested various policy changes to increase the documentation and use of SDOH data, with changes to payment being most frequently mentioned. Across groups, clinicians endorsed making the time they spend discussing SDOH with patients reimbursable as well as providing financial incentives for achieving documentation targets. In addition, many recommended providing education to guide clinicians in how to have SDOH conversations, both in the preclinical period (eg, medical school) and through ongoing professional education and training.

### Clinician and Patient Preferences for SDOH Data Collection

Most clinicians preferred to store the data in the EHR in structured fields and to collect it using a questionnaire, with an option to include free text to capture key information gathered during conversation (Table 3). In terms of when SDOH data should be collected, responses were split. Some clinicians deemed it better to collect before a visit, while others preferred having this done during a visit through staff.

**Table 3.** Practitioner and Patient-Identified Preferences for Future Data Collection

Preference	Examples (group)
Structured, in app	“I would use an app. I don’t know that I feel it’s any less confidential than you using the portal. We run risks all the time with everything we do online. So I don’t really have a problem with an app. As a matter of fact, my portals are on apps already. So I’m using an app for the most part to get on to the portals.” (Patients ≥65 y) “If we could overcome some of the barriers to collecting SDOH data through a 3rd party app, that would be great. As long as the app was straight forward and patient friendly.” (Rural primary care)
Structured, in person	“During the patient visit by providing the patient a means of privately answering the questions so as not to feel uncomfortable or be feared of being discriminated against. I believe this could be accomplished with a simple questionnaire prior to interacting with the physician.” (Emergency medicine) “I feel like I would rather them ask on paper so that way a lot of people will feel more confident in doing it. So a lot of times pride will probably have people say no if you ask them like flat out. I would rather do a kind of discreetly first.” (Parents or guardians of children <18 y)
Structured, patient portal	“I would rather do as little as possible when I get to the office and just you don’t have to get there early, just get there on time for the appointment. I rather do it in a privacy of my own home or I can do it my leisure or sometimes you want to look something up and check it and then you try to look it up when you’re in the office, trying - what is this medication? What’s the doses? I’d rather do it at home and have it follow me to the office and have it done.” (Patients ≥65 y) “Ideally, much of this could be completed pre-visit through patient portals along with specific health concerns that an individual/family wishes to address at the time of the visit. Knowing that pre-visit entry might not work for many patients, it could then be accomplished in the waiting room and added to or modified during the visit.” (Pediatrics)
Conversation	“So I’ve always been comfortable talking to somebody one on one rather than just filling out a form, but not everybody’s like that, so I get it.” (Parents or guardians of children <18 y) “Ideally I would prefer patients share SDOH verbally so that additional questions can be asked when appropriate to do so. I generally prefer gathering this information in a conversational type of manner.” (Case management)

Abbreviation: SDOH, social determinants of health.

Patients similarly preferred data collection through structured questionnaires on paper, the patient portal, or through an app, with discussion during the visit as needed (Table 3). Only participants in the Spanish language group strongly preferred conversation alone. When the questionnaire was conducted through the portal, patients mentioned having more time to generate thoughtful responses to questions and allow their clinician time to review before their appointment. When asked about their willingness to use an app to provide SDOH data, patients expressed concerns about privacy and security of apps as well as app overload. However, they reported being more likely to use an app endorsed by a trusted clinician.

## Discussion

The growing body of evidence on the impact of SDOH on health has spurred an increasing emphasis on the collection of critical data elements for population health management and individual care plans.^36,37^ Our findings from 20 focus groups with 235 patients, care partners, and clinicians suggest several areas to be considered as efforts continue to collect, document, and share this information.

Participants agreed that SDOH data are important to patient care; however, patients were ambivalent about the documentation and sharing of data they considered sensitive, expressing concerns about how the data will be used and how they might be (mis)perceived. Patients wanted to understand why SDOH data were needed as well as how providing these data would help them and to have a say in how this information was shared. This echoes earlier research suggesting some individuals are uncomfortable with having SDOH data in their records.^24,25,26,38^ Patients’ recent experiences with the health care system, including perceived quality of care, trust in the health care system, and experiences with racial and financial discrimination when receiving care, likely also affect their comfort with clinicians sharing these data.^39,40,41^

The development of technical and policy solutions allowing patients more control over how their data are documented and shared is underway, although not imminent. Organizations are seeking to increase support for data segmentation, allowing data to be tagged as sensitive and enabling patients to indicate whether such information would be shared.^42^ However, implementing this change is technically challenging.^43^ Furthermore, segmentation of data in this way could increase care fragmentation, negatively affect quality of care, and increase the number of patients with unmet needs. The goal of providing patients with greater control of their data must be weighed against these risks.

In the near term, greater transparency around SDOH data could help address some of these concerns. Educational and outreach efforts could help to reassure patients that their information will not be used to discriminate against them or limit their care and potentially increase their willingness to share.^44^ Additional curricula during clinical training and continuing education focused on talking with patients about SDOH issues could increase comfort with discussing these sensitive issues, as could enhanced training for clinic staff who may be collecting SDOH information as one of many tasks conducted during the patient check-in process. Developing such skills may also help cultivate a stronger and more trusting clinician-patient relationship—a key mediating factor in patients’ willingness to share SDOH data in our study. For clinicians, resources exist on how to document SDOH data in structured and easily accessible formats and how to retrieve and use the data for population health management.^44^ Clinicians identified significant barriers to both data collection and the ability to discuss these data with their patients, including lack of time and resources, consistent with other studies that found technical, workflow, and financial challenges.^45,46,47,48^ Pilot studies of payment models such as the Making Care Primary model, which supports primary care practices meeting patients’ SDOH needs, are underway and increasingly supported by CMS.^49^ Clinicians participating in such payment models have higher rates of engaging in SDOH-related data collection, indicating the potential impact of these models.^50,51^ CMS has further proposed adjusting quality measures using community-level metrics such as the Area Deprivation Index which could reduce the need for individual data collection while ensuring they still account for SDOH factors in health care payments.^52^

### Limitations

This study has several limitations. First, the findings offer lessons and suggestions but are not generalizable beyond the groups, and traditional validity metrics are not appropriate for RAP. Second, our findings may have been influenced by participants with strong views. We attempted to mitigate this risk by using a standard interview guide and professional moderator. Third, while we recruited participants for the focus groups across a spectrum of specialty, practice type, race, ethnicity, and geography to ensure broad representation, our criteria were not exhaustive and important experiences and views may have been unintentionally excluded.

## Conclusions

As national efforts focus on promoting standardized data collection, this qualitative study of the experience of collecting, documenting, and exchanging SDOH data underscores the ongoing barriers to widespread adoption of uniform approaches to documentation and factors that may help to lower them, including greater transparency and education, strong patient-clinician relationships, and additional financial incentives and resources to allow for greater time with patients and targeted assistance. A multifaceted approach to addressing the issues raised by clinicians and patients in this study is required to ensure that such data can be captured in a way that improves care and allows for progress toward an equitable health care system.

## Notes

Supplement 1. **eAppendix 1.** Environmental Scan Search Terms **eAppendix 2.** Advisory Panel Members **eAppendix 3.** Focus Group Moderator Slides **eAppendix 4.** Focus Group Codes and Themes **eAppendix 5.** Characteristics of Focus Group Participants

Supplement 2.
Data Sharing Statement
