# Supplementary material for: Clinician and Patient Perspectives on the Exchange of Sensitive Social Determinants of Health Information
Source: JAMA Netw Open. 2024 Oct 31;7(10):e2444376. doi: 10.1001/jamanetworkopen.2024.44376 (PMC11528312; doi:10.1001/jamanetworkopen.2024.44376)
Supplement: Supplement 1. — eAppendix 1. Environmental Scan Search Terms eAppendix 2. Advisory Panel Members eAppendix 3. Focus Group Moderator Slides eAppendix 4. Focus Group Codes and Themes eAppendix 5. Characteristics of Focus Group Participants [file jamanetwopen-e2444376-s001.pdf]

Supplemental Online Content

DesRoches CM, Wachenheim D, Garcia A, et al. Clinician and patient perspectives on the exchange of sensitive social determinants of health information. *JAMA Netw Open.* 2024;7(10):e2444376. doi:10.1001/jamanetworkopen.2024.44376

eAppendix 1. Environmental Scan Search Terms

eAppendix 2. Advisory Panel Members

eAppendix 3. Focus Group Moderator Slides

eAppendix 4. Focus Group Codes and Themes

eAppendix 5. Characteristics of Focus Group Participants

This supplemental material has been provided by the authors to give readers additional information about their work.

© 2024 DesRoches CM et al. *JAMA Network Open.*

eAppendix 1. Environmental Scan Search Terms

**Terms Related to Documentation**		**Terms Related to EHR**		**Terms Related to Social Determinants of Health, [illegible]**
unstructured OR unstructured data OR documentation	AND	clinical notes OR notes OR visit notes OR medical record OR electronic health record	AND	social determinants of health
				transportation
				housing
				homelessness
				IPV/interpersonal violence
				employment status
				social risk
				social history
				food insecurity
				housing
				financial stress
				sexual orientation/gender identity
				education
				educational attainment
				employment
				social isolation
				stress
				documentation of stress
				patient experience
				patient perspective
				provider experience
				provider perspective
				physician perspective
				physician experience

© 2024 DesRoches CM et al. *JAMA Network Open.*

eAppendix 2. Advisory Panel Members

Profession	Organization
**Patient Engagement Director; Internist**	Los Angeles Department of Health Services
**Clinical Data Scientist**	National Association of Community Health Centers
**Founder**	Enlightening Results
**Internist**	Beth Israel Deaconess Medical Center
**Senior Practice Specialist**	American Physical Therapy Association
**Managing Director for Health Justice**	National Partnership for Women and Families
**Associate Director; Primary Care Physician**	University of California, San Fransisco Center for Vulnerable Populations; Zuckerberg San Francisco General Hospital
**Managing Director, Digital Health Strategy & Policy**	Savage & Savage LLC
**Community Outreach Specialist**	Dreyfus Health Policy and Research Center
Mental Health Liaison	OpenNotes, Beth Israel Deaconess Medical Center

eAppendix 3. Focus Group Moderator Slides

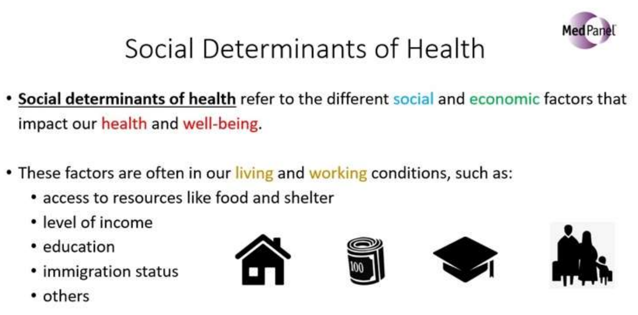

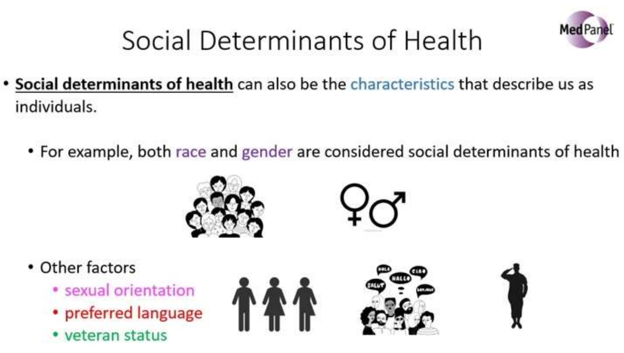

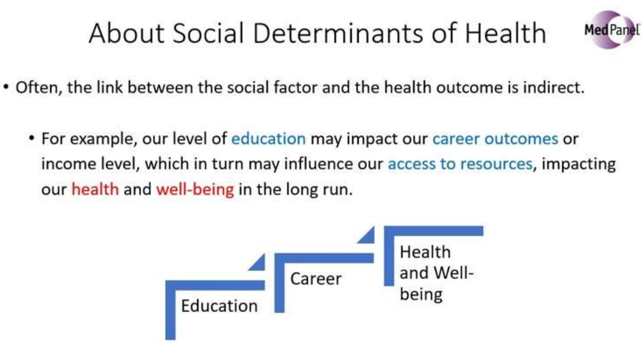

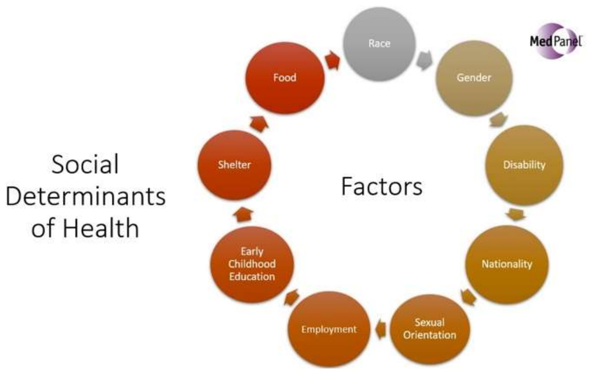

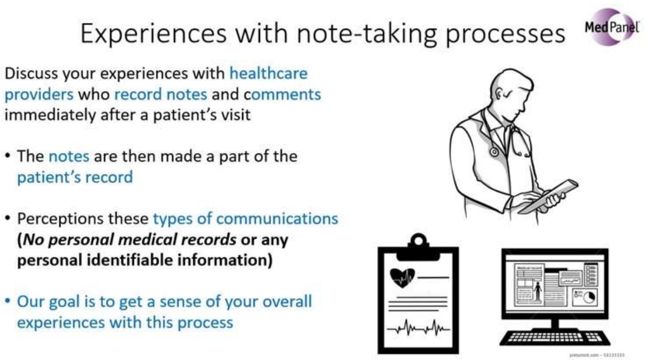

© 2024 DesRoches CM et al. *JAMA Network Open.*

eAppendix 4. Focus Group Codes and Themes

Theme	Sub-theme	Example
Factors Affecting Standardized Social Determinants of Health (SDOH) Data Collection	Beliefs about use	Discomfort with topic as barrier to Social Determinants of Health data exchange for patient
		Worries about discrimination
		Privacy concerns
		Not sure of the value of the information
		Do not trust outside information as barrier to exchanging information
		Cultural differences as barrier to Social Determinants of Health data for clinician
	Relational	Clinician discomfort with topic
		Social Determinants of Health data effect on patient/provider relationship as barrier
		Provider believes patient will be uncomfortable discussing as barrier
		Poor patient/provider relationship as barrier
		Trusting clinician-patient relationship
	Structural	Provider does not want to document because cannot act on the information
		Data collection is not reimbursable
		Time in visit
		Increased community resources
	Workflow	Lack of time in visit to collect and address issues
		Do not have the right staff
	Technology	General technology barriers
		Digital divide – disparities in access
		Difficult to find information in the Electronic Health Record (EHR)
		Portal issues
		Lack of interoperability
		Control over how and with whom data is shared
	Policy	Education for patients and clinicians
		Reimburse time spent in data collection and addressing Social Determinants of Health issues
		Financial incentives
		Insurance or government mandates
Preferences for collecting/sharing Social Determinants of Health	Structured	Paper questionnaire
		Patient portal questionnaire
		App
	Unstructured	Conversation with provider

© 2024 DesRoches CM et al. *JAMA Network Open.*

information in the future		

© 2024 DesRoches CM et al. *JAMA Network Open.*

eAppendix 5. Characteristics of Focus Group Participants

Clinician Demographics	
**Race**	
White	74
Black/African American	2
Asian	25
American Indian or Alaska Native	2
More than 1 race	2
Other	2
Preferred not to answer	2
**Ethnicity**	
Hispanic/Latinx	6
Not Hispanic or Latinx	101
Preferred not to answer	2
**Gender**	
Female	47
Male	61
Not identify as Female, Male or Transgender	1
**Geography**	
Northeast	38
South	22
Midwest	28
West	21

Patient Demographics	
**Race**	
Black/African American	48
White	64
Asian	1
More than 1 race	1
Other	2
**Ethnicity**	
Hispanic/Latin X	17

© 2024 DesRoches CM et al. *JAMA Network Open.*

Not Hispanic/Latin X	99
Gender	
Female	69
Male	45
Transgender	1
Genderqueer/Non-binary	1
Age	
19-29	18
30-34	28
35-39	16
40-44	14
45-49	10
50-54	3
55-59	9
60-64	2
65+	16
Geography*	
Northeast	31
South	47
Midwest	31
West	17

** Geographic data includes e-patients. E-patients are not included in the other demographic group.*

© 2024 DesRoches CM et al. *JAMA Network Open.*
