# Supplementary material for: Clinician and Patient Perspectives on the Exchange of Sensitive Social Determinants of Health Information
Source: JAMA Netw Open. 2024 Oct 31;7(10):e2444376. doi: 10.1001/jamanetworkopen.2024.44376 (PMC11528312; doi:10.1001/jamanetworkopen.2024.44376)
Supplement: Supplement 2. — Data Sharing Statement [file jamanetwopen-e2444376-s002.pdf]

Data Sharing Statement

DesRoches. Clinician and Patient Perspectives on the Exchange of Sensitive Social Determinants of Health Information. *JAMA Netw Open*. Published October 31, 2024. doi:10.1001/jamanetworkopen.2024.44376

Data
Data available: No

Additional Information
Explanation for why data not available: We will make our coding scheme and focus group guides available. We will not make the focus group transcripts available out of concern for participants privacy. Given the small number of participants in each group, it would be relatively easy for a knowledgeable person to identify study participants known to them.
